# Linear and Branched Glyco-Lipopeptide Vaccines Follow Distinct Cross-Presentation Pathways and Generate Different Magnitudes of Antitumor Immunity

**DOI:** 10.1371/journal.pone.0011216

**Published:** 2010-06-21

**Authors:** Olivier Renaudet, Gargi Dasgupta, Ilham Bettahi, Alda Shi, Anthony B. Nesburn, Pascal Dumy, Lbachir BenMohamed

**Affiliations:** 1 Laboratory of Cellular and Molecular Immunology, The Gavin Herbert Eye Institute, School of Medicine, University of California Irvine, Irvine, California, United States of America; 2 Département de Chimie Moléculaire, UMR-CNRS 5250 and ICMG FR 2607, Université Joseph Fourier, Grenoble, France; 3 Institute for Immunology, University of California Irvine Medical Center, Irvine, California, United States of America; 4 Chao Family Comprehensive Cancer Center, University of California Irvine Medical Center, Irvine, California, United States of America; New York University, United States of America

## Abstract

**Background:**

Glyco-lipopeptides, a form of lipid-tailed glyco-peptide, are currently under intense investigation as B- and T-cell based vaccine immunotherapy for many cancers. However, the cellular and molecular mechanisms of glyco-lipopeptides (GLPs) immunogenicity and the position of the lipid moiety on immunogenicity and protective efficacy of GLPs remain to be determined.

**Methods/Principal Findings:**

We have constructed two structural analogues of HER-2 glyco-lipopeptide (HER-GLP) by synthesizing a chimeric peptide made of one universal CD4^+^ epitope (PADRE) and one HER-2 CD8^+^ T-cell epitope (HER_420–429_). The C-terminal end of the resulting CD4–CD8 chimeric peptide was coupled to a tumor carbohydrate B-cell epitope, based on a regioselectively addressable functionalized templates (RAFT), made of four α-GalNAc molecules. The resulting HER glyco-peptide (HER-GP) was then linked to a palmitic acid moiety, attached either at the N-terminal end (linear HER-GLP-1) or in the middle between the CD4+ and CD8+ T cell epitopes (branched HER-GLP-2). We have investigated the uptake, processing and cross-presentation pathways of the two HER-GLP vaccine constructs, and assessed whether the position of linkage of the lipid moiety would affect the B- and T-cell immunogenicity and protective efficacy. Immunization of mice revealed that the linear HER-GLP-1 induced a stronger and longer lasting HER_420–429_-specific IFN-γ producing CD8^+^ T cell response, while the branched HER-GLP-2 induced a stronger tumor-specific IgG response. The linear HER-GLP-1 was taken up easily by dendritic cells (DCs), induced stronger DCs maturation and produced a potent TLR- 2-dependent T-cell activation. The linear and branched HER-GLP molecules appeared to follow two different cross-presentation pathways. While regression of established tumors was induced by both linear HER-GLP-1 and branched HER-GLP-2, the inhibition of tumor growth was significantly higher in HER-GLP-1 immunized mice (*p*<0.005).

**Significance:**

These findings have important implications for the development of effective GLP based immunotherapeutic strategies against cancers.

## Introduction

Aberrant glycosylation leads to the expression of abnormal tumor associated carbohydrate antigens (TACAs) and are considered as a unique target for tumor-specific IgG/IgM antibodies [1], [2], [3], [4], [5]. The most common TACA derived B-cell epitope is Tn antigen (a precursor of Thomsen-Friedenreich or TF antigen), also known as GalNAc [5], [6]. Tn-specific IgG/IgM are detected in up to 90% of human carcinomas, but their level and affinity is weak [3]. When administered alone, TACAs activate the antibody secreting B cells weakly [3], [7]. Similarly, tumor-specific CD8^+^ T cells are also detected in cancer patients, but their level and function are not sufficient enough to control tumor progression [6]. Therefore, immunotherapeutic vaccines that can boost the induction of tumor-specific CD8^+^ T cells and boost their function are required for tumor protection. An ideal immunotherapeutic cancer vaccine should comprise both TACA derived carbohydrate B-cell epitope and tumor associated antigen (TAA) -derived CD8^+^ T-cell epitopes to boost the sub-optimal antitumor B- and T cell immune responses often detected in cancer patients [8], [9], [10], [11].

Molecularly defined and human compatible self-adjuvanting vaccines that are capable of inducing tumor-specific antibody and CD8^+^ T-cell immunity are limited (reviewed in [1] and [12]). The low molecular weight lipid molecule (palmitic acid) is derived from a immunologically active lipoprotein of *Escherichia coli* origin [1], [9] and has been widely used, as an adjuvant, to enhance the immunogenicity of both peptide T-cell epitopes [9], [13], [14], [15], [16], [17], [18] and carbohydrate B-cell epitopes [19], [20], [21]. Palmitic acid (PAM) also acts as a biological ligand for toll receptor 2 (TLR-2) that is expressed on the surface of antigen presenting cells, such as dendritic cells, [1], [18], [22], [23] and enhances their phenotypic and fuctional maturation [1], [18], [22]. Dendritic cells cross-present exogenous palmitic acid-tailed peptide epitopes (i.e. lipopeptides), associate them with their MHC class I molecules, and present them to prime CD8^+^ T cells [1], [9], [21], [24], [25]. Two major routes for cross-presentation of lipid-tailed molecules have been described: (*i*) processing in the cytosol as they exit from the endocytic compartment [26], [27], [28]; and (*ii*) processing in the endosome and transfer of the peptides to recycling MHC class I molecules either in the endocytic pathway or, after regurgitation, at the cell surface [26], [27], [28]. We have recently reported that immunization of mice with an ovalbumin GLP vaccine construct (OVA-GLP) induced B- and T-cell dependent protective immunity, in both therapeutic and prophylactic settings [1], [21], [24]. However, the GLP vaccine strategy has never been extended to relevant TAA-derived epitopes. In addition, whether the position of the lipid moiety into GLP molecules affects the processing and presentation of T cell and B cell epitopes as well as their in vivo immunogenicity has never been investigated.

In this study, we have constructed a prototype HER-2 glyco-lipopeptide (HER-GLP) cancer vaccine by incorporating all the necessary components, including TACA as B cell epitopes, CD4^+^ and CD8^+^ T cell peptide epitopes and an internal immuno-adjuvant (palmitic acid) in one construct in order to boost potent and specific antitumor B and T cell immunity. We studied how the position of the lipid moiety (i.e. either at the N-terminal end or in the middle of the GLP molecule) affects the uptake of HER-GLP by DCs, and the processing and cross-priming pathways that lead its functional presentation to CD8+ T cells. Our results show that the position of lipid moiety not only affected the uptake and cross-presentation pathways of GLP in DCs but, interestingly, modulated the magnitude of antitumor antibody and CD8+ T-cell protective immunity. These findings have considerable implications for GLP vaccine development.

## Results

### Design and assembly of prototype multivalent B, CD4+ and CD8+ epitopes HER-2 glyco-lipopeptide molecules

We designed two prototype HER-2 glyco-lipopeptide molecules: (*i*) a linear HER-GLP-1 molecule and (*ii*) a branched HER-GLP-2 molecule produced using the chemoselective strategy based on a combined oxime/disulfide bond formation. Both GLP vaccine molecules were regioselectively assembled and contained: (*i*) one CD8+ T-cell epitope peptide (DSLRDSVF) from HER-2; (*ii*) one universal CD4+ T-helper epitope (AKXVAAWTLKAAA), known as PADRE; (*iii*) a B-cell epitope made of Regioselectively Addressable Functionalized Templates (known as RAFT molecules), which represented a cluster of Tn (α-GalNAc)_4_) carbohydrate antigen analogues; and (*iv*) one palmitic acid moiety, which plays the role of internal immuno-adjuvant. The resulting PAM-HER_420–429_-PADRE-RAFT is designated as “HER-GLP”. The corresponding non-lipidated structural analog HER_420–429_-PADRE-RAFT is designated as “HER-GP”.

**Figure 1:**
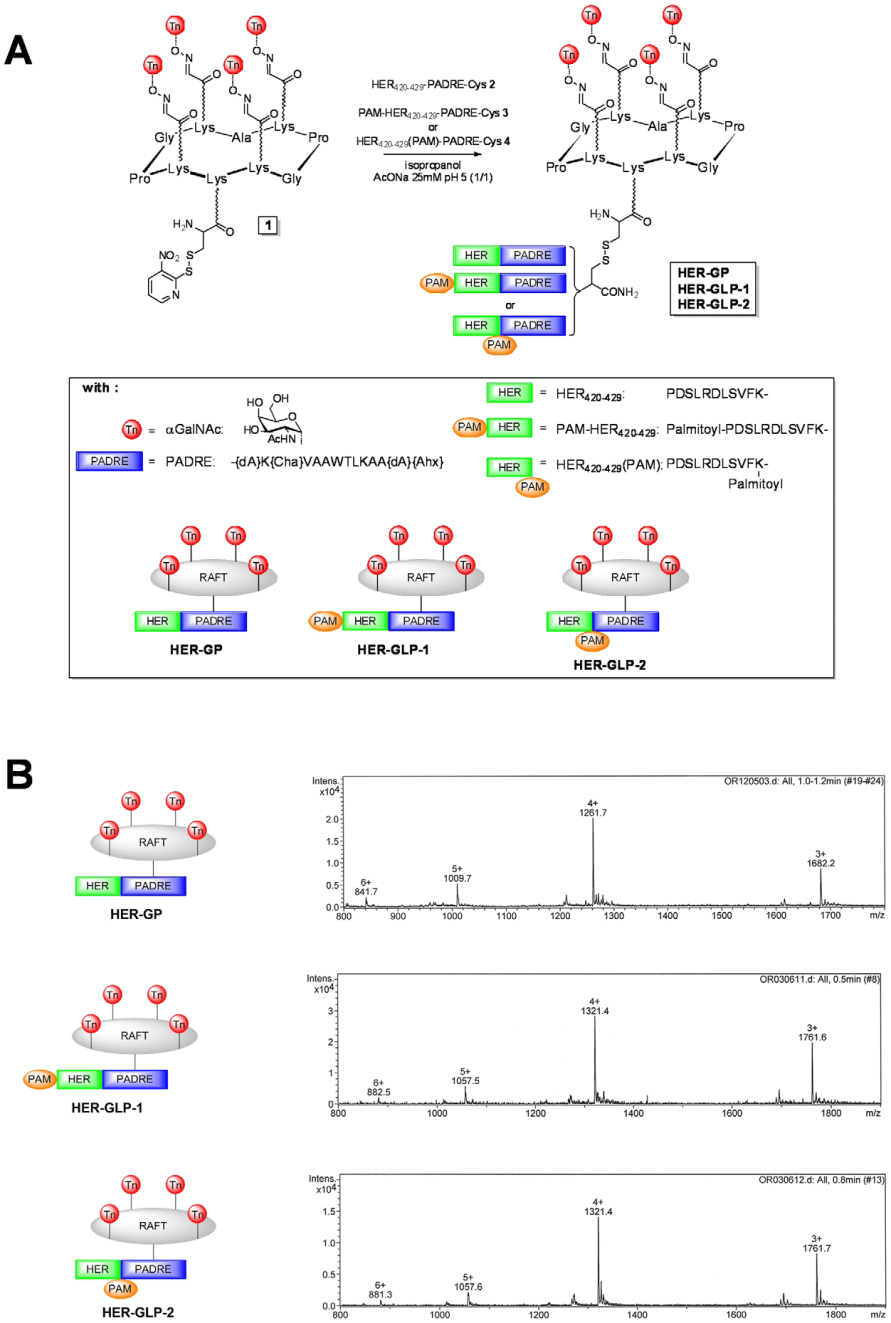
Assembly, structures and mass spectrum analyses of prototype multivalent B, CD4+ and CD8+ epitopes HER-2 glyco-lipopeptide molecules. (**A**) The RAFT moiety **1** was assembled from an orthogonally protected linear decapeptide following the standard Fmoc/tBu strategy, as we previously described [21]. The amino acid sequences of CD4^+^ and CD8^+^ epitopes and cyclic template are given using one letter code. Unusual amino acids are designated as dA (L-alanine), Cha (cyclohexyl alanine) and Ahx (L-2-aminohexanoic acid). Each compound displays clustered Tn analog on the upper domain of the cyclodecapeptide RAFT template. HER-GP and HER-GLP contain respectively either HER_420–429_-PADRE chimeric peptide or PAM- HER_420–429_-PADRE chimeric lipopeptide on the lower domain. (**B**) Mass spectrum (MS) analysis was obtained by electron spray ionization (ESI-MS) in the positive mode. The multi-charged ions observed for HER-GP (*m/z*: 841.7 [M+6H]^6+^, 1009.7 [M+5H]^5+^, 1261.7 [M+4H]^4+^, 1682.2 [M+3H]^3+^), HER-GLP-1 (*m/z*: 882.5 [M+6H]^6+^, 1057.5 [M+5H]^5+^, 1321.4 [M+4H]^4+^, 1761.6 [M+3H]^3+^) and HER-GLP-2 (*m/z*: 881.3 [M+6H]^6+^, 1057.6 [M+5H]^5+^, 1321.4 [M+4H]^4+^, 1761.7 [M+3H]^3+^) correspond to the expected deconvoluated masses calculated for [M+H]^+^ (5044.3 for HER-GP, 5282.8 for HER-GLP-1 and 5282.9 for HER-GLP-2).

Linear HER-GLP-1, branched HER-GLP-2 and non-lipidated HER-GP were assembled from compound 1 using convergent ligation chemistry based on oxime and disulfide bond formation described earlier (Fig. 1A) [1], [21], [24]. The cyclopeptide RAFT scaffold 1 displays the cluster of Tn antigen to ensure efficient antigen delivery and contains a cystein-Npys moiety on the other addressable domain. This activated cysteine residue permits the conjugation of peptide or lipopeptide-containing cysteine at the C-terminal end. The final disulfide coupling reaction between compound 1 (Fig. 1A) and the peptide HER_420–429_-PADRE 2 or lipopeptides PAM-HER_420–429_-PADRE 3 and HER_420–429_-(PAM)-PADRE 4 was performed under argon gas in a mixture of isopropanol and sodium acetate buffer. The purified HER-GP glyco-peptide, the linear HER-GLP-1; and the branched HER-GLP-2 glyco-lipopeptides were homogeneous in solution and their expected primary molecular weights were derived by electrospray mass spectrometry (Fig. 1B). Multiple freeze-thaw cycles, over a period of one year, did not disrupt the physicochemical properties of the HER-GLP vaccine in solution [29]. Finally, the linear HER-GLP-1, the branched HER-GLP-2 and their parent non-lipidated HER-GP were labeled unequivocally by fluorescence probe Alexa Fluor 488 to study their entry into immature dendritic cells by confocal microscopy.

### Branched HER-GLP-2 induced stronger RAFT-specific IgGs than linear HER-GLP-1

**Figure 2:**
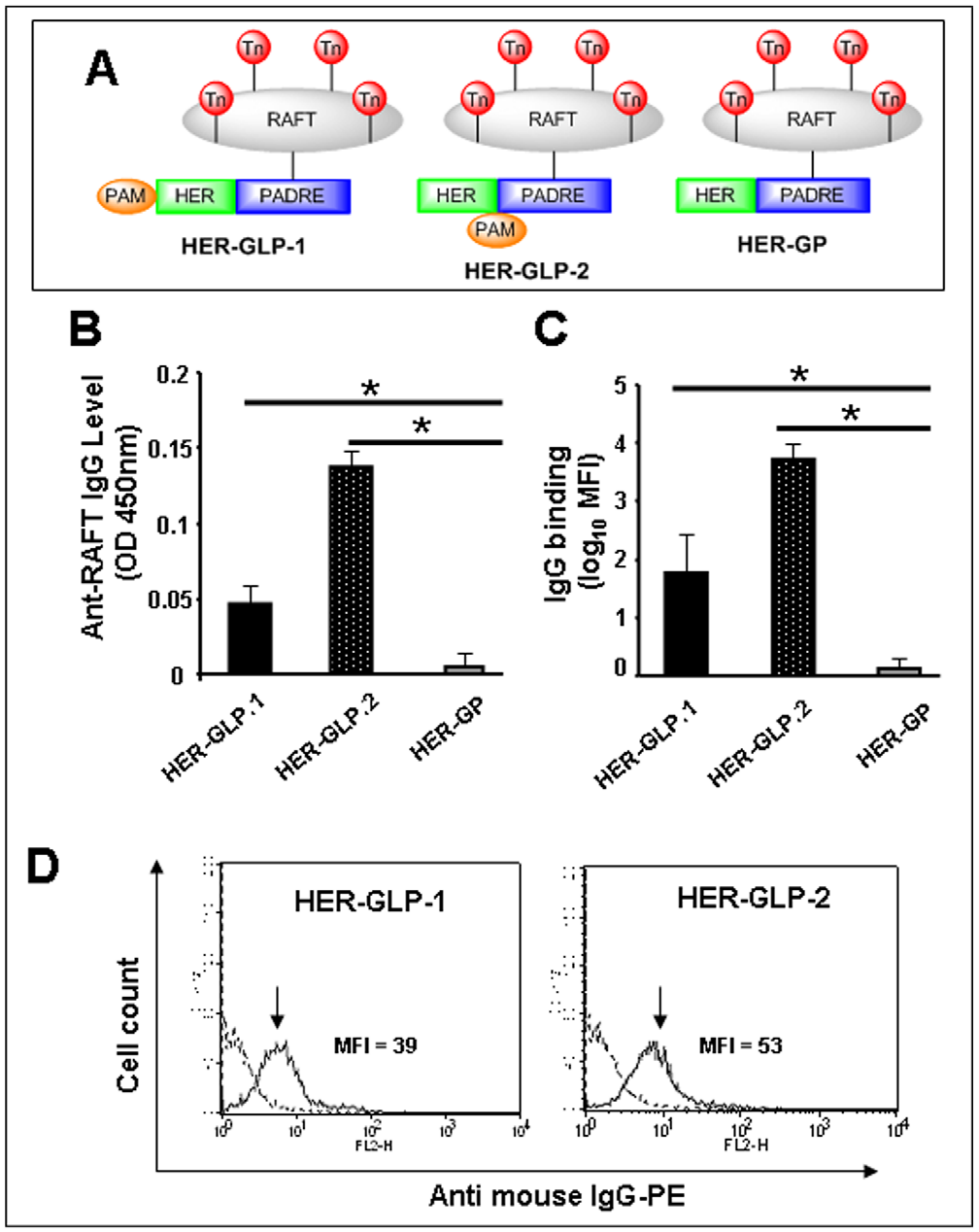
Immunization with branched HER-GLP-2 induces stronger RAFT-specific IgGs than linear HER-GLP-1. Three groups of B10.D1 mice (*n* = 10) were immunized subcutaneously two times with an interval of 14 days, with linear HER-GLP-1 (50 µM/mouse) or branched HER-GLP-2 (50 µM/mouse) or non-lipidated HER-GP (50 µM/mouse) as shown in (panel **A**) or injected with PBS alone. Ten days after the 2^nd^ immunization, serum was collected from each mouse and the level of RAFT-specific IgG (panel **B**) was measured by ELISA. MCF7 cells (4×10^5^ cells) were incubated for 30 min with 10 µl of mice sera from HER-GLP-1, HER-GLP-2, HER-GP immunized or PBS-injected control mice (Mock) at 1∶250 dilutions and analyzed by flow cytometry using FITC labeled goat anti mouse IgG antibody. The binding efficiency was calculated in terms of mean fluorescent intensity (MFI) after subtracting the background of serum binding from mock-immunized mice (panel **C**). A representative data showing the binding of serum from HER-GLP-1 and HER-GLP-2 immunized mice (solid lines) and serum from mock-immunized mice (broken lines) with MCF7 cells (panel **D**). The results are representative of three experiments.

To compare the B-cell immunogenicity of linear HER-GLP-1 and branched HER-GLP-2 molecules (Fig 2A), B10.D1 mice (10 mice/group) were immunized subcutaneously with equimolar amount of either linear HER-GLP-1 (Group 1) or branched HER-GLP-2 (Group 2) vaccine constructs in adjuvant-free saline. A third group of ten B10.D1 mice was immunized with the non-lipidated HER-GP analog in adjuvant-free saline (Group 3) and used as control. A fourth group of ten B10.D1 mice was injected subcutaneously with saline alone (Group 4, Mock). No adverse reaction such as local inflammation at the sites of injection or weight loss was observed in any immunized mice rendering the safety of these adjuvant-free vaccine formulations. Ten days after the 2^nd^ immunization, the serum IgG levels specific to carbohydrate RAFT were determined in each group by ELISA. Significant levels of RAFT-specific IgG were induced in both linear HER-GLP-1 and branched HER-GLP-2 immunized mice ((Fig. 2B) *p*<0.05 and *p*<0.01, respectively). However, the branched HER-GLP-2 appeared as a better immunogen than the linear HER-GLP-1, suggesting that the position of the lipid moiety affects the magnitude of antibody responses generated by the GLP vaccines. Interestingly, immunization of mice with non-lipidated HER-GP did not induce any significant level of RAFT-specific IgG responses (*p*>0.05), revealing the requirement for the built-in palmitic acid in generating carbohydrate specific IgG Abs. Constructs missing any of the four components did not produce Abs response suggesting that it is crucial to have all the components linked within one molecule (*not shown*).

The IgGs induced by both linear HER-GLP-1 and branched HER-GLP-2 construct bind human breast tumor cell line MCF7 expressing Tn molecules (Fig. 2C and 2D). However, higher (350 fold) binding was observed for branched HER-GLP-2 induced IgGs than linear HER-GLP-1 induced IgGs (150 fold) when compared to non-immune control IgGs (*p*<0.01). Under identical experimental conditions, IgGs induced by both HER-GLP-1 and HER-GLP-2 immunogen did not show any binding to T2 and RS cell lines that do not express Tn molecules (*not shown*).

Taken together, these results indicate that while both linear HER-GLP-1 and branched HER-GLP-2 molecules induced RAFT-specific IgGs that bind to human tumor cell lines expressing the native Tn antigen, the branched HER-GLP-2 appeared to be a stronger B-cell immunogen than the linear HER-GLP-1.

### Linear HER-GLP-1 induced stronger and long lasting HER_420–429_-specific IFN-γ-producing CD8+ T cell responses than branched HER-GLP-2

**Figure 3:**
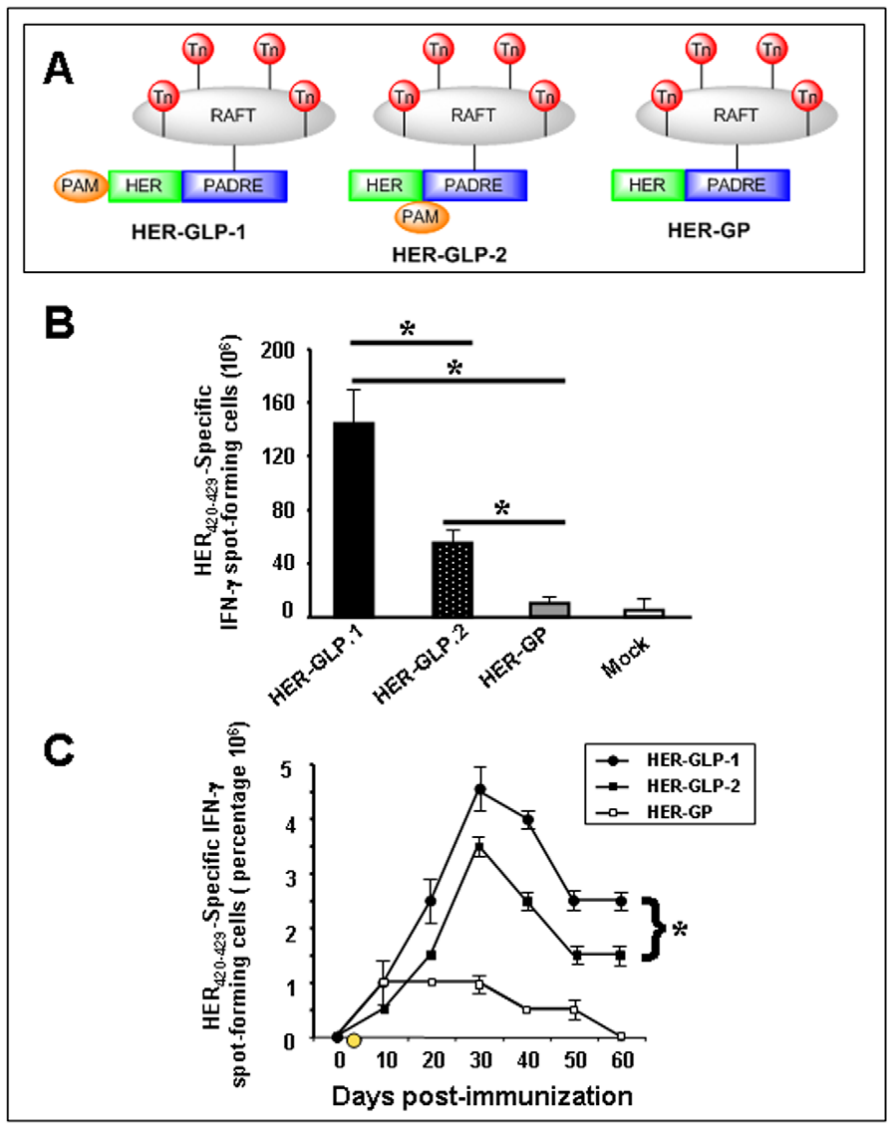
Immunization with linear HER-GLP-1 induces stronger and long-lasting HER_420–429_-specific IFN-γ producing CD8+ T cell responses than branched HER-GLP-2. Three groups of B10.D1 mice (*n* = 10) were immunized subcutaneously three times with HER-GLP-1 (50 µM/mouse), HER-GLP-2 (50 µM/mouse) or HER-GP (50 µM/mouse) as shown in panel **A**) or injected with PBS alone (Mock) with an interval of 14 days. Ten days after the 3^rd^ immunization, spleen cells were isolated and stimulated *in vitro* for 4 days with HER_420–429_ peptide and assayed for IFN-γ producing CD8+ T cells by ELISpot. Mean values (± SD) of IFN-γ spot-forming CD8+ T cells were plotted against each group of mice and are shown in (**B**). Kinetics of HER_420–429_-specific IFN-γ producing CD8+ T cells were measured in mice immunized with HER-GLP-1, HER-GLP-2 and HER-GP from 0 to 60 days of post immunization and is shown in (**C**). The results are representative of three experiments.

Groups of B10.D1 mice (10/group) were immunized subcutaneously three times at fourteen-day intervals with equimolar amount of linear HER-GLP-1 or branched HER-GLP-2 (Fig 3A). As a control a third group of ten B10.D1 mice was immunized with the non-lipidated HER-GP analog. A fourth group of ten B10.D1 mice was injected subcutaneously with saline alone (*Mock*). Ten days after the third immunization, HER_420–429_-specific CD8+ T cell responses were evaluated in the spleen.

Spleen-derived cells were re-stimulated *in vitro* with HER_420–429_ peptide for four days and HER_420–429_-specific IFN-γ producing CD8+ T cell responses were measured by ELISpot assays. As shown in Fig 3B, both linear HER-GLP-1 and branched HER-GLP-2 immunized mice developed significant number of HER_420–429_-specific IFN-γ producing CD8+ T cells when compared with mock-immunized control mice (*p*<0.05). However, the linear HER-GLP-1 showed higher number of HER_420–429_-specific IFN-γ producing CD8+ T cells than the branched HER-GLP-2, suggesting that the position of the lipid moiety in the GLP construct does affect the magnitude of CD8+ T cell responses. As expected, the HER-GP immunized mice showed a non-significant number of HER_420–429_-specific CD8+ T cells when compared to mock-immunized control mice (*p*>0.05), further underlining the requirement for the built-in palmitic acid immuno-adjuvant for the induction of the T-cell responses. Constructs missing one of the four components or non-covalent mixtures of the parts did not induce a T cell response suggesting that it is crucial to have all the components linked within one molecule (*not shown*).

We next performed a kinetic measurement of HER_420–429_-specific IFN-γ-producing CD8+ T cells in mice immunized with both linear HER-GLP-1 and branched HER-GLP-2 for up to 60 days post immunization. We observed that the percentage of HER_420–429_-specific IFN-γ-producing CD8+ T cells was gradually increased and reached the peak 4.5% for HER-GLP-1 and 3.5% for HER-GLP-2 on day 28 (Fig. 3C). Thereafter, the percentage of HER_420–429_-specific IFN-γ-producing CD8+ T cells were gradually decreased but instead of completely declining, a certain percentage (2.5% for HER-GLP-1 and 1.8% for HER-GLP-2) of HER_420–429_-specific IFN-γ-producing CD8+ T cells were maintained up to day 60. Taken together, these results indicate that while both linear HER-GLP-1 and branched HER-GLP-2 molecules are capable of inducing a long-lasting HER_420–429_-specific CD8+ T-cell response, the linear HER-GLP-1 appeared to be a stronger T-cell immunogen than the branched HER-GLP-2. These results illustrate the importance of the position of palmitic acid moiety on HER-GLP vaccine construct in terms of maintaining the long lasting IFN-γ-producing CD8 T cells. Unlike the CD8+ T cell responses, PADRE-specific CD4+ T cell proliferative responses were not affected by the position of the lipid moiety (*not shown*).

### Regression of established tumors following immunotherapeutic immunization with linear HER-GLP-1 and branched HER-GLP-2 vaccines

**Figure 4:**
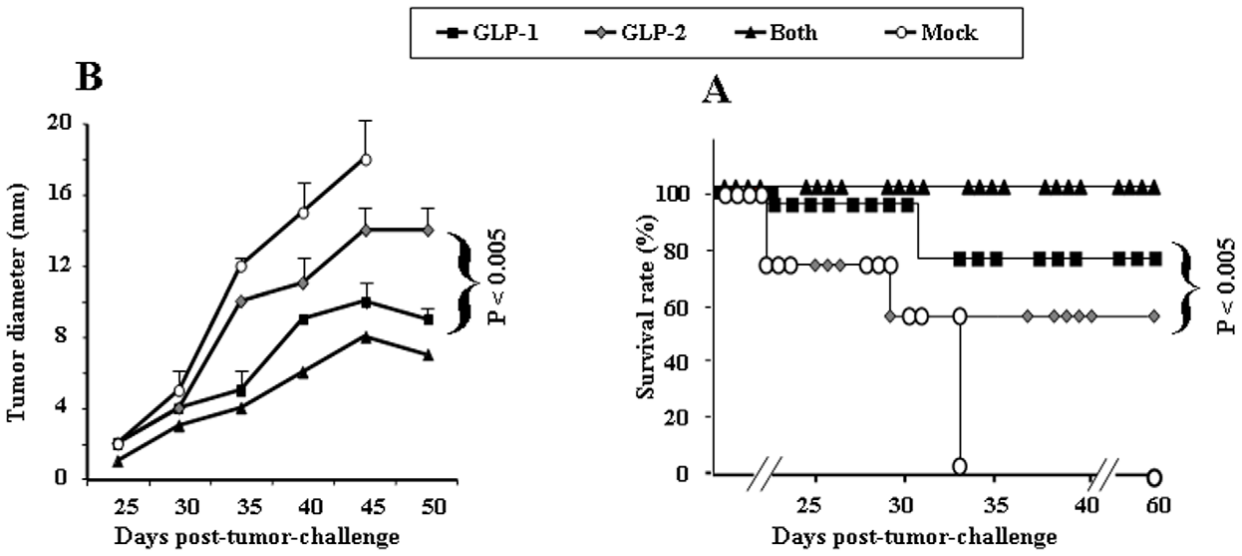
Immunotherapeutic efficacy of linear HER-GLP-1 and branched HER-GLP-2 vaccine constructs. NT2 cells (1×10^5^/mouse) were injected s.c. in the mammary fat pad of 40 female B10.D1 mice (5 wk old). Eight days later, when tumor diameter reached 3–4 mm, mice were divided into 4 groups of 10 mice each. Group 1 was immunized s.c. four times at seven day intervals with the self-adjuvanting linear HER-GLP-1 (*GLP-1*), group 2 was immunized with the branched HER-GLP-2 (*GLP-2*), group 3 was immunized with both HER-GLP-1 and HER-GLP-2 (*both*), and group 4 was injected with PBS alone as control (*Mock*). (**A**) **Tumor progression.** Local tumor dimensions were measured with calipers as described in *Materials and Methods*. The average of tumor diameters (in millimeters) in the course of 50 days is presented. (**B**) **Survival.** Mice from the same experiment were monitored daily for 90 days and were sacrificed when moribund, which corresponded to a tumor diameter of 18 mm. The results are presented as mean+SEM. Both **A** and **B** present *p* values calculated to compare the two groups of HER-GLP-1 and HER-GLP-2 immunized mice (i.e. group 1 and group 2). Data are representative of two independent experiments.

The immunotherapeutic efficacy of self-adjuvanting linear HER-GLP-1 and branched HER-GLP-2 vaccine molecules were compared by assessing tumor growth and mice survival rate. To develop tumor, female B10.D1 mice (10 per group) were implanted subcutaneously in the mammary fat pad with 1×10^5^ NT2 cells. Eight to ten days later, when tumor diameter reached 3–4 mm, mice were immunized subcutaneously four times at seven day intervals with the linear HER-GLP-1 (group 1), the branched HER-GLP-2 (group 2), both HER-GLP-1 and HER-GLP-2 (group 3) or injected with PBS alone (group 4, mock). As shown in Fig. 4A, the tumor diameter (mm), which reflects the tumor progression, was significantly delayed in mice vaccinated with the linear HER-GLP-1 molecules compared with mock-immunized mice (*p*<0.005). However, therapeutic immunization with HER-GLP-2 did not lead to a significant reduction the tumor progression (p>0.005). The inhibition of tumor growth was significantly higher in linear HER-GLP-1 compared to branch HER-GLP-2 immunized mice (*p*<0.005). Interestingly, the protective efficacy detected in mice immunized with both HER-GLP-1 and HER-GLP-2 (group 3) was slightly higher than mice immunized with individual HER-GLP-1 and HER-GLP-2 constructs. The strong immunotherapeutic effect of the linear HER-GLP-1 was also evident from the survival graph (Fig. 4B). Of 10 mice vaccinated with the linear HER-GLP-1 and the branched HER-GLP-2 molecules, 8 of 10 and 6 of 10 were still alive over 8-week after tumor inoculation, respectively. Therapeutic immunization with both HER-GLP-1 and HER-GLP-2 protected 10 of 10 mice from death as compared to 0 of 10 mock-immunized mice (*p*<0.005).

### Cytoplasmic uptake of linear HER-GLP-1 and branched HER-GLP-2 constructs by dendritic cells

**Figure 5:**
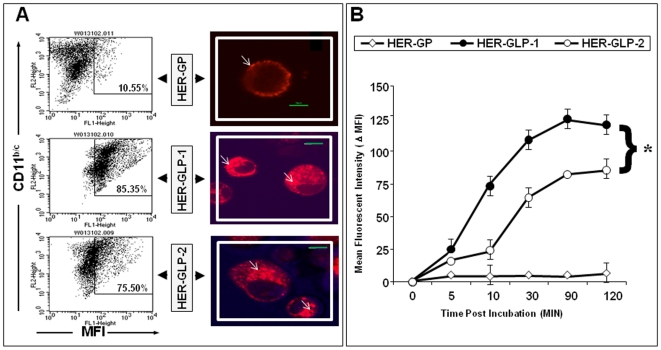
Relative uptake of linear HER-GLP-1; branched HER-GLP-2 and non-lipidated HER-GP molecules by bone marrow derived immature dendritic cells. (**A**) Primary cultures of bone marrow derived DC populations were incubated for 30 min at 37°C with Alexa Fluor 488-labeled HER-GLP-1, HER-GLP-2 or HER-GP at an equimolar concentration of 1 uM each. Left panel shows the dot plot representation of loaded HER-GLP and HER-GP constructs on CD11b/c^+^ cells and right panel shows the subsequent cytoplasmic localization of HER-GLP and HER-GP constructs by confocal microscopy. (**B**) Shows the uptake kinetics of HER-GLP and HER-GP constructs by CD11b/c^+^ cells following incubation at different time intervals as measured by the endocytosis assay uptake. Endocytosis assay uptake of Alexa Fluor 488-labeld HER-GP and HER-GLPs by DCs was determined for 120 min both at 4C° and 37C°. Subsequently, DCs were washed three times with phosphate-buffered saline/bovine serum albumin 1% and total uptake of Alexa Fluor 488-labeld was measured by FACS analyses and expressed by the difference in geometric mean that resulted from subtracting the values obtained at 4C° from the values obtained at 37C°. This formula determines the amount of HER-GP and HER-GLPs that is actively internalized. The results are representative of five experiments.

In an effort to elucidate the mechanisms underlying the immunogenicity of linear HER-GLP-1 and branched HER-GLP-2 molecules, we determined the kinetics of their uptake by immature dendritic cells (DCs). Mouse bone marrow derived immature DCs were incubated with equimolar amount of Alexa Fluor 488 labeled HER-GLP-1 or HER-GLP-2 or non-lipidated HER-GP constructs. The uptake of each vaccine construct on DCs surface was analyzed by FACS and their cytoplasmic accumulation was visualized by confocal microscopy. Both linear HER-GLP-1 and branched HER-GLP-2 were efficiently taken up by DCs at a concentration as low as 1 uM (Fig. 5A left panel). Cytoplasmic accumulation of both linear HER-GLP-1 and branched HER-GLP-2, but not HER-GP, was visualized within 10 min of incubation (Fig. 5A, right panel). The Alexa Fluor 488-labeled HER-GP was unable to cross DC membrane even after several trials at higher concentration. This suggests that the attachment of a palmitic acid moiety play an important role in the entry of HER-GLP constructs into the cytoplasm of DCs.

Endocytosis assay uptake showed a total active uptake of HER-GP and HER-GLPs was measured by FACS and expressed by the difference in geometric mean of delta fluorescence intensity (Δ MFI) that resulted from subtracting the values obtained at 4C° from the values obtained at 37C°. Kinetic studies showed that the linear HER-GLP-1 was quickly taken up at 37°C by DCs within 10 minutes, whereas the uptake of branched HER-GLP-2 was relatively slower (Fig. 5B). Upon longer incubation at 37°C, the mean ΔMFI of the linear HER-GLP-1 construct on DCs was significantly higher than the branched HER-GLP-2 (*p*<0.05). After 30 min incubation, up to 85% of DCs were associated with the linear HER-GLP-1 and about 75% of DCs were associated with the branched HER-GLP-2 analog (Fig. 5A). A gradual increase in the Δ MFI of both constructs associated with DC was observed over time, reaching a plateau by 90 min (Fig. 5B). However, no further changes in the Δ MFI were observed between 90 to 120 min of incubation. The cytoplasmic entry of both linear HER-GLP-1 and branched HER-GLP-2 occurred at 37°C but was inhibited at 4°C (*not shown*), indicating an active intracellular delivery mechanism. Together, these results show that both the linear HER-GLP-1 and branched HER-GLP-2, but not the non-lipidated HER-GP analog, were taken up quickly by the DCs and accumulated into DCs cytoplasm. The uptake of linear HER-GLP-1 appeared to be relatively faster compared to the branched HER-GLP-2 analog, suggesting that the position of the lipid moiety might affect the uptake of GLP constructs by DCs.

### The linear HER-GLP-1 construct induced stronger dendritic cell maturation and followed by toll-like receptor 2-dependent T-cell activation

**Figure 6:**
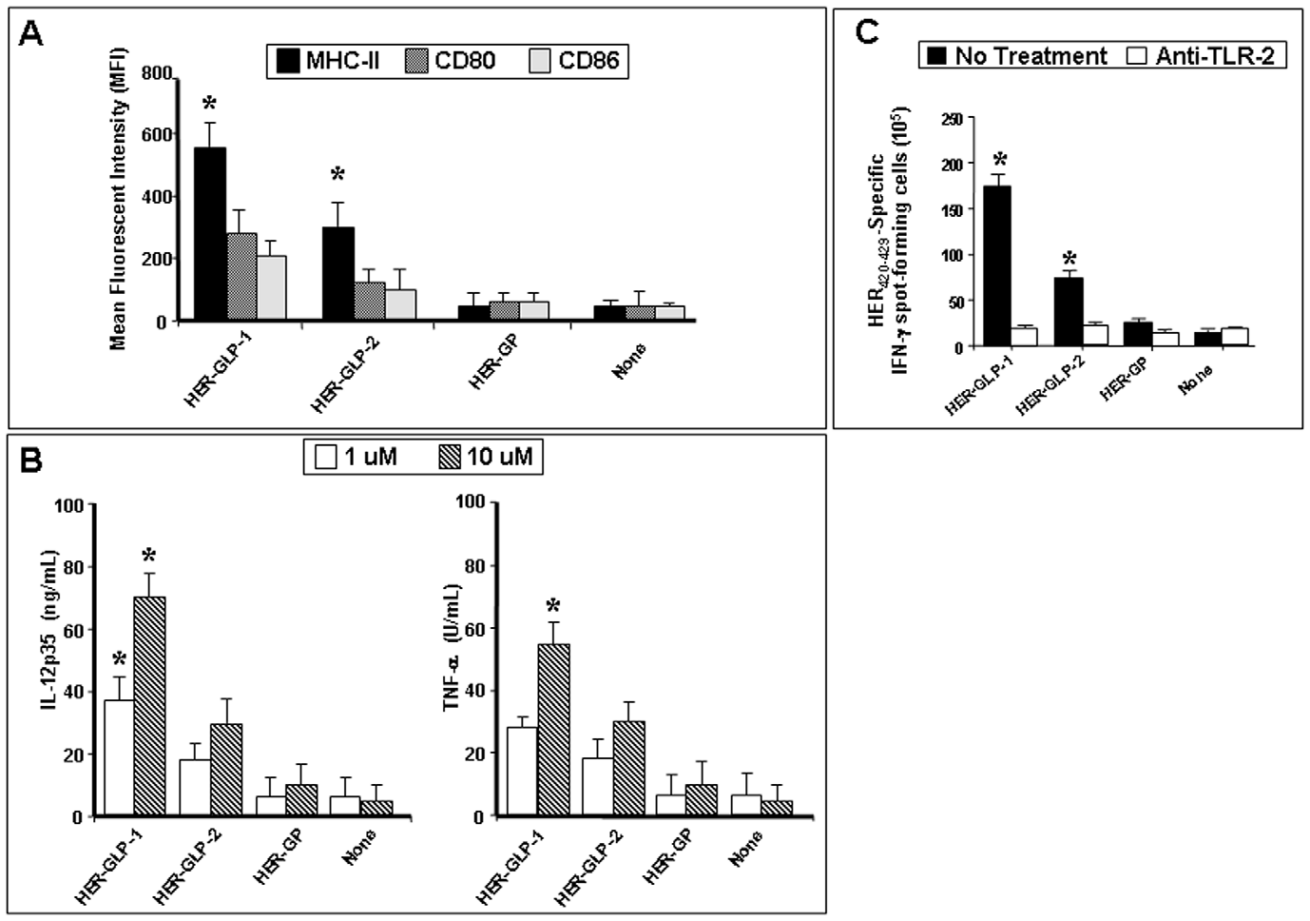
Phenotypic and functional maturation of dendritic cells induced by linear HER-GLP-1, branched HER-GLP-2 and HER-GP molecules. (**A**) Immature DCs were derived from mouse bone marrow and either left untreated (none) or incubated in vitro for 48 hrs with equimolar amount of linear HER-GLP-1, branched HER-GLP-2, or non-lipidated HER-GP analog. Phenotypic markers for DC maturation (major histocompatibility complex (MHC) class II, CD80, CD86) were analyzed by FACS and plotted in terms of calculated MFI. (**B**) IL-12p35 and TNF-α released by linear HER-GLP-1, branched HER-GLP-2 and HER-GP induced matured DCs were measured by cytokine assay, as described in *Materials and Method*. Panel (**C**) shows the Inhibition of HER_420–429_-Specific IFN-γ spot-forming CD8+ T cells by anti TLR2 antibody as described in *Materials and Method*. The results are representative of three experiments.

Next we sought to determine whether HER-GP and HER-GLP constructs are capable of inducing DC maturation and whether the position of the lipid moiety affects such maturation. Immature DCs were derived from mouse bone marrow and left untreated (none) or incubated in vitro for 48 hrs with an equimolar amount of either linear HER-GLP-1 or branched HER-GLP-2, or non-lipidated HER-GP and monitored the expression of cell surface major histocompatibility complex (MHC) class II, and B7 (CD80 and CD86) co-stimulatory molecules which are the well known phenotypic markers for DC maturation. Incubation of immature DCs with either linear HER-GLP-1 or branched HER-GLP-2 constructs induced significant up-regulation of MHC class II, CD80 and CD86 co-stimulatory molecules compared with non-lipidated HER-GP construct (Fig. 6A). In addition, the incubation of immature DCs with either linear HER-GLP-1 or branched HER-GLP-2 was associated with an increase in the production of IL-12p35 and TNF-α cytokines (Fig. 6B; *P*<0.005) in a concentration dependent manner. Under similar conditions, immature DCs incubated with the non-lipidated HER-GP construct failed to up-regulate the DC markers of maturation and did not produce the pro-inflammatory cytokines. These data suggest that the covalent linkage of the lipid moiety not only facilitated the uptake of the vaccine constructs but also supported phenotypic maturation of DCs.

In a separate ELISpot assay, we measured the number of HER420_-429_-specific IFN-γ producing CD8^+^ T-cells in the presence of bone marrow derived autologous immature DCs pre-pulsed with equimolar amount of linear HER-GLP-1 or branched HER-GLP-2 or non-lipidated HER-GP. Higher numbers of IFN-γ-secreting HER_420–429_-specific CD8^+^ T-cells were detected after stimulation with linear HER-GLP-1-pulsed DCs compared to stimulation with the branched HER-GLP-2-pulsed DCs (*p*<0.05) (Fig. 6C). None of the HER_420–429_-specific CD8+ T-cells were activated following incubation with immature DCs alone (none) or with DCs pulsed with a control irrelevant HSV-1 gB_495–505_ peptide (not shown).

To determine whether toll receptors (TLR-2 and TLR-4) on DCs is playing a potential role in stimulating HER_420–429_-specific CD8+ T-cells by linear HER-GLP-1 and branched HER-GLP-2 constructs, blocking experiments were performed. We incubated autologous immature DCs with either anti-TLR-2 or anti-TLR-4 mAbs, for 30 min before pulsing with the equimolar amounts of either linear HER-GLP-1, branched HER-GLP-2 or non-lipidated HER-GP. The anti-TLR-2 mAbs, but not the anti-TLR-4 mAbs, significantly abrogated the production of the IFN-γ by HER_420–429_-specific CD8+ T-cells (*p* = 0.002) indicating that both linear HER-GLP-1 and branched HER-GLP-2 activate DCs *via* a TLR-2-dependent pathway (Fig. 6C). Collectively, these results show that the phenotypic maturation of DCs induced by the linear HER-GLP-1 and branched HER-GLP-2 occurred through the TLR-2 signaling pathway.

### The position of the lipid moiety profoundly affects the cross-presentation pathway of glyco-lipopeptides

To determine the cross-presentation pathways of HER-GLP-loaded DCs, we used specific antigen-processing inhibitors: brefeldin A, epoxomycin and monensin. Brefeldin A inhibits passage from the endoplasmic reticulum to the Golgi, the exocytic pathway [30] or inhibits the level of MHC class I molecule recycling [31]. Epoxomycin acts as a specific proteasome inhibitor [32] and inhibits the chymotrypsin-like activity and to a lesser extent the trypsin-like and peptidyl-glutamyl peptide-hydrolyzing activities of the proteasome. Epoxomycin is very specific for the proteasome and does not inhibit non-proteasomal proteases such as trypsin, chymotrypsin, papain, cathepsin B, calpain, or tripeptidyl peptidase II [33]. The internalization of exogenous antigen by endocytosis and subsequent processing by DCs may occur through the endosomal pathway [27]. Monensin inhibits endosomal acidification, enzymatic degradation in the lysosomal compartments and as such might disturb endocytosis [27], [34].

**Figure 7:**
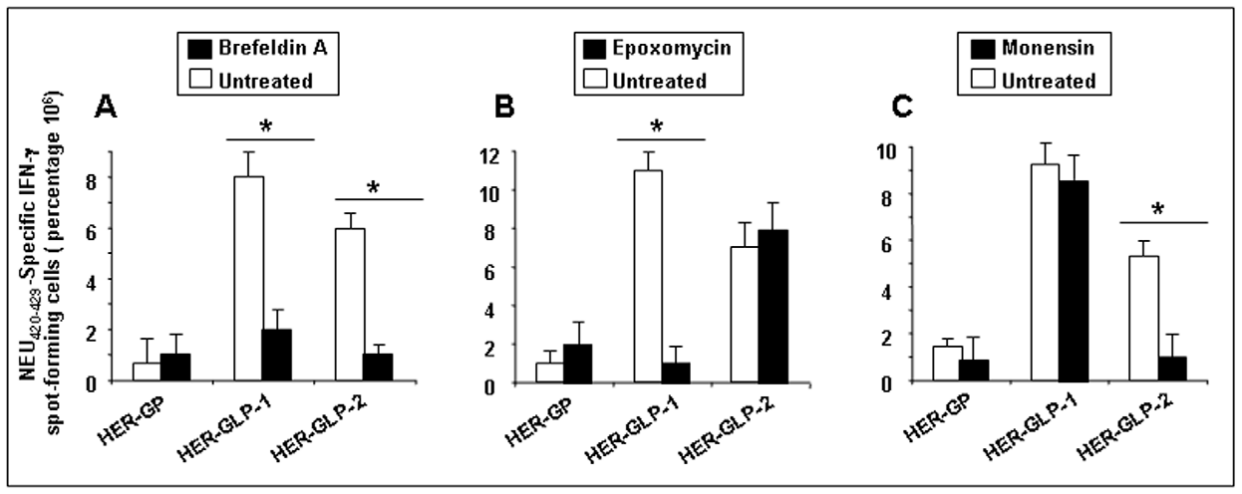
Cross-presentation pathways of linear HER-GLP-1 and branched HER-GLP-2 vaccine constructs in dendritic cells. (**A**) Dendritic cells were pre-incubated with brefeldin A for 1 h, followed by the addition of linear HER-GLP-1, branched HER-GLP-2 or non-lipidated HER-GP construct (*open bars*). As a positive control, DCs were left untreated with antigen-processing inhibitors but were pulsed with linear HER-GLP-1, branched HER-GLP-2 or parent non-lipidated HER-GP (*open bars*). After overnight incubation, DCs were then washed and added to the HER_420–429_-specific CD8^+^ T cells for additional 5 hrs. IFN-γ produced by HER_420–429_-specific CD8^+^ T cells was tested by ELISpot assay. Panel (**B**) and (**C**) represents identical experiments conducted in the presence of epoxomycin and monensin inhibitors, as described in *Materials and Methods*. Results are representative of three independent experiments.

To assess the cross-presentation pathway, dendritic cells were first treated with brefeldin A, Epoxomycin or Monensin, as described in *Materials and Methods*, followed by the addition of linear HER-GLP-1, branched HER-GLP-2 or parent non-lipidated HER-GP construct. After overnight incubation, DCs were washed and added to the HER_420–429_-specific CD8^+^ T cells for additional 5 hrs. As a positive control, DCs were left untreated with antigen-processing inhibitors but were pulsed with linear HER-GLP-1, branched HER-GLP-2 or parent non-lipidated HER-GP constructs. HER_420–429_-specific CD8^+^ T cell responses were detected by ELISpot, as above. As shown in Fig. 7A, brefeldin A significantly inhibited the magnitude of IFN-γ-producing HER_420–429_-specific CD8^+^ T-cells induced by both the linear HER-GLP-1 and branched HER-GLP-2 constructs. This indicates that the antigen processing of both the linear HER-GLP-1 and branched HER-GLP-2 is governed by the passage from the endoplasmic reticulum to the Golgi. As shown in Fig. 7B, epoxomycin almost completely blocked the presentation of the linear HER-GLP-1 to HER_420–429_-specific CD8+ T cells. This indicated that the HER-GLP-1 is processed by the proteasome, which led to effective presentation of the HER_420–429_ epitope and stimulation of a stronger IFN-γ-producing HER_420–429_-specific CD8^+^ T-cells. In contrast to linear HER-GLP-1, the branched HER-GLP-2 was still processed and presented in the presence of epoxomycin (Fig. 7B). This result shows that processing of branched HER-GLP-2 and presentation of HER_420–429_ epitope to CD8+ T cells is independent on the proteasome pathway. An opposite result was observed when monensin was used as the inhibitor (Fig. 7C). Monensin significantly inhibited the presentation of branched HER-GLP-2 but not the linear HER-GLP-1 indicating that at least partial processing of the branched HER-GLP-2 in the endosomal compartment and/or recycling through monensin-sensitive endocytic vesicles. Conversely, presentation of the linear HER-GLP-1 was not inhibited, suggesting that neither a monensin-sensitive endogenous pathway nor endosomal acidification were used during the processing of linear HER-GLP-1 construct.

## Discussion

Glyco-lipopeptides, a form of lipid-tailed glyco-peptides, are currently under intense investigation as B- and T-cell based vaccine immunotherapy for many cancers [35], [36], [37], [38], [39]. In the present report, we describe the assembly, immunogenicity and antitumor efficacy of four-component HER-GLP vaccine construct (one CD4^+^ T-cell epitope, one OVA CD8^+^ T-cell epitope, one carbohydrate B-cell epitope, and a built-in palmitic acid adjuvant). The additional incorporation of a palmitic acid moiety in two different positions results in linear and branched constructs termed HER-GLP-1 and HER-GLP-2, respectively. We showed that both constructs are immunogenic. While the linear HER-GLP-1 induces more potent HER_420–429_-specific IFN-γ-producing CD8^+^ T cell responses, the branched HER-GLP-2 promotes stronger tumor-specific IgG responses. Furthermore, although both constructs enter dendritic cells (DCs) via TLR2 and induced DCs maturation each construct appeared to be processed and presented to T cells differently. Accordingly, therapeutic immunization of mice with linear HER-GLP-1 versus branched HER-GLP-2 induced different levels of antitumor protective immunity. Thus, the position of the lipid moiety within synthetic GLP vaccine constructs greatly influence: (*i*) the magnitude of induced IgG and CD8+ T cell responses; (*ii*) the phenotypic and functional maturation of DCs; (*iii*) the cross-presentation pathway of the GLP constructs by DCs; and (*iv*) the level of therapeutic efficacy against established tumors.

This work demonstrate that the linear HER-GLP-1 construct induced higher magnitude of epitope-specific IFN-γ-producing CD8^+^ T-cell response while the branched HER-GLP-2 analogue preferentially induced stronger IgG antibody response. Besides promoting a stronger CD8+ T cell response, the self-adjuvanting linear HER-GLP-1 also induced higher-level phenotypic and functional maturation of dendritic cells. Finally, we established that the uptake of linear HER-GLP-1 construct did not follow the classical cross-presentation pathway as evidenced by the endosome-to-cytosol inhibition study, but rather appeared to take the proteasomal pathway. The molecular mechanisms that led to the exit of the epitopes from endosome to cytosol remain to be determined. To our surprise, the processing pathway of branched HER-GLP-2 was different from the linear HER-GLP-1 construct. The processing of branched HER-GLP-2 was not sensitive to epoxomycin suggesting that the branched HER-GLP-2 construct is processed through a proteasome-independent pathway. The branched HER-GLP-2 processing is, however, sensitive to both brefeldin A and monensin suggesting the processing is dependent on lysomal acidification as well as on MHC class I recycling and transport from endosome to cell surface. To our knowledge, this is the first report that the position of the lipid moiety within a GLP vaccine construct greatly affects its cross-presentation pathway resulting in modulation of the magnitude of induced B and T-cell responses.

The processing of linear HER-GLP-1 is not sensitive to monensin suggesting that the pathway is independent of lysosomal acidification. As the processing of linear HER-GLP-1 is sensitive to both brefeldin A and epoxomycin it suggests that after uptake by DCs the construct was processed through proteasome mediated digestion followed by loading of the HER_420–429_ epitope on MHC class I molecules. In the endosomal system, the linear HER-GLP-1 may be protected from the predominant cleavage by the proteasome that would destroy the HER_420–429_ epitope in the cytosol and may find a suitable enzyme for optimal generation and presentation of this epitope. However, the identity of this enzyme remains to be determined. Association with MHC class I molecules is possible in late endosomes, which were found to contain MHC class I molecules [40]. Alternatively, the linear HER-GLP-1 might also egress to the cytosol from a different, monensin-sensitive compartment than the branched HER-GLP-2 analogue and be digested by an enzymatic activity that is insensitive to epoxomycin. Finally, we do not exclude that the affinity of the chemical bond between the palmitic acid moiety and the peptide backbone might be slightly different during the synthesis of the linear HER-GLP-1 versus the branched HER-GLP-2. This might play a role in determining how the GLP is taken-up and processed in DCs, as recently demonstrated using the palmitoylated encephalitogenic peptides of myelin proteolipid protein [41].

Our results demonstrated that both linear HER-GLP-1 and branched HER-GLP-2 constructs are taken up easily by dendritic cells (Fig. 5), and both constructs induced DCs maturation, albeit at different levels (Fig. 6A and 6B). Processing of both GLP constructs by DCs, which leads to different magnitudes of T cell stimulations (Fig. 3 and Fig. 7), appeared to involve binding/internalization *via* TLR-2 molecules. This is supported by our antibody blocking experiment, where blocking TLR-2, but not TLR-4, abrogated the presentation of CD8+ T cell epitope to HER_420–429_-specific IFN-γ-producing T cells (Fig. 6C). The position of the TLR-2 ligand palmitic acid appeared to influence the cross-presentation pathway of GLP vaccine constructs within DCs, and this might be a consequence of a difference in binding/internalization process *via* TLR-2. Recent study reported that peptides linked to TLR-2 ligand Pam(3)Cys of R-configuration (Pam(R)) lead to better activation of DCs compared to those with S-configuration (Pam(S)) [42], [43]. Although both Pam(R) and Pam(S) epimers were internalized equally, the study concluded that the enhanced DC maturation is due to enhanced TLR-2 binding by the Pam(R)-conjugate in contrast to its Pam(S)-conjugate. Similarly, in case of linear and branched HER-GLP constructs, one cannot exclude the possibility of two different affinities of palmitic acid with TLR2, when placed in two different chemical conformations, cause differential uptake/processing in DCs [44]. Our results certainly show that the position of TLR-2 ligand palmitic acid, (i.e. linear or branched) greatly affects the uptake and the cross-presentation pathway of associated epitopes. In addition, the involvement of other TLRs and non-TLR receptors are not ruled out in the binding/internalization of linear HER-GLP-1 and branched HER-GLP-2 [45]. Another TLR-2 ligand lipoteichoic acid was reported be internalized independently from TLR2 [46]. Investigating the relative role of TLRs and other non-TLR receptors in: (*i*) binding/internalization; (*ii*) processing; and (*iii*) enhancing the immunogenicity and protective efficacy of GLPs is an important goal for future studies. Because each of the GLP constructs employed in the present study is molecularly defined, they can be labeled precisely on a single residue for future mechanistic evaluation.

Production of CD8^+^ T cell epitopes in APCs has been mostly documented as processing by the proteasome in the cytosol followed by TAP-mediated transport into the endoplasmic reticulum and association with nascent MHC class I molecules [40], [47]. Among APCs, only DCs can prime naive CD8^+^ T cells, and therefore they are required for primary immunization [40], [48], [49], [50]. Dendritic cells have apparently evolved specific cross-presentation mechanisms allowing them to prime CD8^+^ T cells for exogenous Ags that are first internalized by macro-pinocytosis, phagocytosis, or receptor-mediated endocytosis [31], [51], [52], [53]. Dendritic cells process these antigens and associate them with their MHC class I molecules [52]. Two major routes for cross-presentation have been described [31], [53], [54]: exit from the endocytic compartment and processing in the cytosol [55], [56] and processing in the endosomal system and transfer of the peptides to recycling MHC class I molecules either in the endocytic pathway [57], [58] or, after regurgitation, at the cell surface [59], [60]. These mechanisms are essential for the cells to become sensitive to and develop tolerance to Ags that are not endogenously synthesized in DCs.

Cancer cells undergo significant changes in carbohydrate expression (aberrant glycosylation), and these alterations can be used as therapeutic targets (reviewed in [1]). Aberrant glycosylation of glycoproteins and glycolipids on tumor cells leads to expression of abnormal tumor associated carbohydrate antigens (TACAs) [61], [62]. The most common TACA is Tn antigen (a precursor of Thomsen-Friedenreich or TF antigen), also known as GalNAc, which is α-linked to a serine or threonine residue (α-GalNAC-*O*-Ser/Thr) [63]. Tn is detected in up to 90% of human breast, ovary, and colon carcinomas [64]. However, the induction of IgG antibodies (Abs) against TACAs is much more difficult than eliciting similar Abs against pathogen associated carbohydrates antigens (reviewed in [1]). This is not surprising, because some TACAs are self-antigens and are consequently tolerated by the immune system [3], [65], [66], [67], [68]. The shedding of TACAs by growing tumors exacerbates this tolerance [3], [69], [70]. However, under appropriate conditions, Tn can induce tumor-specific IgG in both mice and non-human primates [29]. Overall the rates of Tn expression showed statistically significant differences between healthy and tumorous or transitional tissues [71], [72]. Accordingly, several Tn-based vaccines and immunotherapies that passed clinical trials showed no major side effects [73]. This raises the hope that aberrantly glycosylated TACAs can be used as a specific target for humoral-mediated cancer vaccines.

The studies reported here show that four-component HER-GLP vaccine molecules elicit robust IgG antibody response. The position of the lipid moiety significantly affects the level of IgG production and the optimal binding to breast cancer cell lines expressing carbohydrate antigens on their surface [20]. Previous studies demonstrated that lipid-tailed peptides promote a T cell-independent activation and maturation of B-cells via TLR-2 and increased the frequency of IgG secreting B-cells [74]. Although B cells expressing TLR-2 are potential targets, very little is known about the effect of GLP on B-cells and especially on their potential for inducing carbohydrate-specific Ab response. Our findings demonstrate that the branched HER-GLP-2 promoted strong Tn carbohydrate-specific IgG response. The same Abs bind to intact Tn expressing breast tumor cells, suggesting that biologically relevant specificities were produced. Although we found that PADRE-specific CD4+ T cell proliferative responses were not affected by the position of the lipid moiety (*not shown*), this does not ruled out the alteration of balance between Th1/Th2 helper cytokines. Why the branched HER-GLP-2 skewed towards stronger RAFT-specific IgG production than linear HER-GLP-1 remains to be determined. Nevertheless, our findings are important, particularly in view of the problems associated with large carrier proteins, such as tetanus toxoid or diphtheria toxoid, often used to deliver weakly antigenic molecules such as carbohydrates [75]. In addition, “booster” injections are often required for conversion of the initial, transient IgM response to a strong, durable IgG response. However, Ab responses directed against the vaccine carrier have been shown, in some cases, to negatively affect the booster response to the vaccine Ag [76]. The use of a totally synthetic GLP carrier molecule capable of inducing vigorous helper T cells, but potentially less readily recognized by Abs might be, in this respect, of significant interest. Together these data support our belief that GLP should be considered as an alternative to more complex carriers for use in prophylaxis and therapeutic cancer vaccines.

The cellular and molecular mechanisms underlying the immunogenicity of HER-GLP remain to be fully elucidated. Our data indicate that the lipid moiety is a crucial factor since the immunogenicity of HER-GLP is superior to its non-lipidated HER-GP analog. Non-lipidated HER-GP failed to cross cell membranes, failed to induce maturation of DCs, and failed to present the HER_420–429_ peptide epitope to CD8^+^ T cells. In addition, vaccine constructs missing any individual component out of the four did not produce any immune responses suggesting that it is crucial to have all the components linked together in one molecule. In contrast, lipid-tailed HER-GLP vaccine molecules easily attach and cross-the cell membrane of DCs and drive their phenotypic and function maturation, and induced a long-lasting presentation of the B- and T-cell epitopes *in vivo* [13], [25], [77]. This point is of crucial importance, because the time elapsing between binding of synthetic peptides and engagement with T cell precursors in secondary lymphoid organs may well exceed the peptide life span at the MHC binding groove, especially for low- to medium-affinity peptides [78]. The use of lipid-tailed HER-GLP appears to partially overcome this shortcoming by endowing the antigenic peptide with longer MHC half-life [23], [77], [79], [80], [81], [82].

It has become increasingly clear that induction and modulation of T cell immunity against tumors require immunogenic formulations that allow efficient targeting and maturation of dendritic cells (DCs) [83], [84]. Dendritic cells are the professional Ag-capturing and Ag-presenting cells with a unique ability to prime naïve T-cells [85]. Dendritic cells have the unique ability to present on MHC class I not only peptides from their own endogenous Ags, but also TAA from their external environment through a process called cross-presentation [86]. After acquiring these TAA, DCs carry this information to secondary lymphoid tissues and present derived epitopes to naive CD8^+^ T cells in ways that initiate immune responses [10], [87]. Critical to this function is a program of maturation that enhances DCs Ag-presenting and costimulatory capacity [85]. Immature DCs were left untreated or incubated *in vitro* with either the lipid-tailed HER-GLPs, the non-lipidated HER-GP or the lipid moiety alone and then monitored for the expression of cell surface MHC class II, CD80 and CD86 co-stimulatory markers indicative of DC maturation. Untreated immature DCs and lipid moiety treated DCs were used as negative controls. Under similar conditions, there was no up-regulation of MHC class II and costimulatory molecules or production of IL-12 or TNF-α following incubation of immature DCs with either the parental non-lipidated HER-GP alone or the lipid moiety alone (Fig. 6A and 6B, *p*<0.05). Non-lipidated HER-GP failed to cross cell membranes and failed to induce phenotypic and function maturation of DCs. In contrast, the lipid-tailed HER-GLPs easily attached and crossed the cell membrane of DCs and drive their maturation. The linear HER-GLP-1 construct was taken up more efficiently by DCs than the branched HER-GLP-2 analog. The present report thus extends previous findings, by showing that *in vitro* incubation of immature DCs with HER-GLP molecules interacts with TLR-2 [37] and increased cell surface expression of MHC class II and CD80/CD86 co-stimulatory molecules resulting in mature DCs producing high levels of IL-12p35 and TNF-α cytokines [1], [21], [24]. Together, these results indicated that covalent linkage to the lipid moiety is required for DCs maturation. This suggests that, as a mechanism behind the immunogenicity of GLPs, the lipid moiety likely exerts its adjuvant effect by interacting and stimulating DCs.

Synthetic cancer vaccines, offer safety, reliability and cost advantages over traditional methods (e.g. live vectors, tumor cells-APC fusions, genetic immunization), but formidable challenges still confront their development [21], [88], [89]. Among these is the requirement for external immuno-adjuvant, which is critical for the immunogenicity and protective efficacy of synthetic vaccines [90], [91]. An ideal adjuvant should rescue and increase the immune response against tumors, with acceptable toxicity and safety even for those immuno-compromised cancer patients [21], [90]. While several different adjuvants are effective in pre-clinical studies, the aluminum-based salt (Alum) is currently the only licensed adjuvant [92], [93] for clinical application. Although “alum” is able to induce a strong antibody (Th_2_ type) response, it has little capacity to stimulate cellular (Th_1_ type) immune responses, which are important for protection against many cancers [8], [94]. Therefore, safe and effective self-adjuvanting molecules are highly desired [3], [21]. These self-adjuvanting cancer vaccine molecules should be more potent but less toxic than external adjuvants [95], [96]. Owing to the limited success of many vaccines in the clinic, attempts are being made to improve the safety and efficacy of vaccine formulations, and to define new adjuvants and antigen delivery systems. However, the development of new cancer adjuvants as well as the improvement of efficacy and safety of existing adjuvants still lags far behind [92], [97]. We have previously shown that the “new generation” four-component, self-adjuvanting GLP vaccine molecule, might offer a compromise between highly toxic adjuvants and no chemical adjuvants at all, while inducing a strong protective immunity [1], [21], [24]. In this study we showed the protective efficacy of a GLP vaccine molecule and demonstrated that the therapeutic efficacy of HER-GLP is affected by the position of the lipid moiety.

Bearing in mind the particular constraints for a prospective human vaccine, the present study designed prototype self-adjuvanting HER-GLPs vaccines and demonstrated their safety, immunogenicity and protective efficacy in a mouse tumor model. Molecularly defined epitope-based vaccines capable of inducing anti-tumor protective immunity, in a manner compatible with human delivery, are limited. Few molecules achieve this target without being delivered with potentially toxic external immuno-adjuvants. It is important to note that even moderate levels of IFN-γ-producing CD8 T cell responses were induced by HER-GLP vaccine constructs (Fig. 3), they were sufficient to control tumor progression (Fig. 4A) and to protect against death (Fig. 4B). This suggests that the quality, rather than the quantity, of the CD8 T cell responses was more crucial in protecting against cancer in this mouse model. We have previously shown that immunization with MHC I-restricted CTL peptides+helper peptides in IFA induced higher magnitude of CTL responses compared to when the same MHC I-restricted CTL peptides+helper peptides are attached to a lipid moiety (i.e. lipopeptides), delivered without external immuno-adjuvant [9], [13]. However, unlike external toxic Freund's adjuvants [98], the TLR-2 ligand palmitic acid, has been used as a built-in immuno-adjuvant, and was safe and immunogenic in both animals and humans [13], [14], [16], [17], [85], [99], [100]. Because several lipid-tailed peptide vaccine candidates have been recently employed in clinical trials [101], [102], [103], we expect that, once a potent lipid-tailed GLP cancer vaccine is validated in pre-clinical animal studies, the move to a clinical trial should be straightforward.

A synthetic liposomal ErbB2/HER2 peptide-based vaccine construct with the combination of CD8^+^ and CD4^+^ epitopes has been recently reported to induce prophylactic and therapeutic protection in mice [104] but concerns remain about its potentially toxic adjuvants. Those studies prompted us to construct and test self-adjuvanting HER-GLP vaccines. Kieber-Emmons and coworkers showed Pam_3_CSS moiety serves as a built-in adjuvant and enhances tumor-specific Abs [22]. Later a three-component GLP vaccine by Boons and coworkers (a three palmitic acid Pam_3_-CysSK_4_ moiety, a CD4+ and a B-Cell epitope) showed induction of strong tumor-specific IgG responses [3]. To our knowledge, we are the first to report self-adjuvanting four-component HER-GLP vaccine molecules. The previously reported complex Pam_3_-CysSK_4_ and Pam_3_CSS molecules have a spontaneous tendency to form stable aggregates, making the synthesis, purification and solubility of Pam_3_CSS-tailed HER-GP extremely difficult. In contrast, our HER-GLP vaccine constructs were synthesized using the mono-palmitoyl strategy which is relatively simple to produce and easy to purify under GMP conditions.

In summary, we have demonstrated that fully synthetic self-adjuvanting linear and branched HER-GLP vaccine molecules follow different cross-presentation pathways in DCs and generate different magnitudes of B- and CD8^+^ T-cell responses. The position the lipid moiety in the HER-GLP construct profoundly affects phenotypic and functional maturation of DCs, the processing of GLP molecules and its cross-presentation in DCs; as well as the magnitude of IgG and IFN-γ producing CD8^+^ T cell responses. Finally, the position of the lipid moiety also affected the strength of immunotherapeutic efficacy induced by the GLP vaccine constructs. The advantage and relative ease of making these self-adjuvanting GLP cancer vaccines will greatly facilitate the production of GLP cancer vaccines for large-scale clinical trials. Their clinical success will depend in great measure on selection of the appropriate human TAAs and TACAs epitopes.

## Materials and Methods

### Peptide, glyco-peptide and glyco-lipopeptides synthesis

#### Peptides

Protected amino acids and Fmoc-Gly-Sasrin resin were obtained from Advanced ChemTech Europe (Brussels, Belgium), Bachem Biochimie SARL (Voisins-Les-Bretonneux, France) and France Biochem S.A. (Meudon, France). PyBOP was purchased from France Biochem and other reagents were obtained from either Aldrich (Saint Quentin Fallavier, France) or Acros (Noisy-Le-Grand, France). Reverse phase HPLC analyses were performed on Waters equipment. The analytical (Nucleosil 120 Å 3 *m*m C_18_ particles, 30×4.6 mm^2^) was operated at 1.3 mL/min and the preparative (Delta-Pak 300 Å 15 *m*m C_18_ particles, 200×25 mm^2^ for glyco-peptides and Discovery® Bio Wide Pore C_5_, 25cm×10 mm) at 22 mL/min with UV monitoring at 214 nm and 250 nm using a linear A-B gradient (buffer A: 0.09% CF_3_CO_2_H in water; buffer B: 0.09% CF_3_CO_2_H in 90% acetonitrile). Mass spectra were obtained by electron spray ionization (ES-MS) on a VG Platform II in the positive mode.

#### Glyco-peptides

Compound **1** [21], [24] (14 mg, 5.89 µmol) was dissolved in a degazed mixture of sodium acetate buffer 25 mM pH 5 and isopropanol (6 mL, 1/1). The peptide HER_420–429_-PADRE-Cys **2** (16.6 mg, 5.89 µmol) was added and the solution was stirred until completeness of the reaction (checked by analytical RP-HPLC). After 2 h, the crude yellow reaction mixture was purified by semi-preparative RP-HPLC (C_18_ column, linear gradient: 95∶5 to 0∶100 A∶B in 30 min, R_t_ = 13.8 min) to obtain a lyophilized powder corresponding the pure glyco-peptide HER-GP (8 mg, 27% yield). Analytical data: R_t_ = 8.2 min (C_18_ analytical column, linear gradient: 95∶5 to 0∶100 A∶B in 15 min); ES-MS (positive mode) calcd. for C_221_H_361_N_58_O_72_S_2_ 5043.6, found 5044.3.

#### Linear glyco-lipopeptide (HER-GLP-1)

A similar procedure was followed from 1 (15 mg, 6.31 µmol) and the lipopeptide PAM-HER_420–429_-PADRE-Cys 3 (19.3 mg, 6.31 µmol) for the synthesis of the glyco-lipopeptide HER-GLP-1. After purification by semi-preparative RP-HPLC (C_5_ column, linear gradient: 95∶5 to 0∶100 A∶B in 30 min, R_t_ = 22.5 min) the glyco-lipopeptide HER-GLP-1 was obtained as pure lyophilized powder (6 mg, 18% yield). Analytical data: R_t_ = 11.8 min (C_18_ analytical column, linear gradient: 95∶5 to 0∶100 A∶B in 15 min), ES-MS (positive mode) calcd. for C_237_H_391_N_58_O_73_S_2_ 5281.8, found 5282.8.

#### Branched glyco-lipopeptide (HER-GLP-2)

A similar procedure was followed from **1** (15 mg, 6.31 µmol) and the lipopeptide PAM-HER_420–429_-PADRE-Cys **4** (19.3 mg, 6.31 µmol) for the synthesis of the glyco-lipopeptide HER-GLP-2. After purification by semi-preparative RP-HPLC (C_5_ column, linear gradient: 95∶5 to 0∶100 A∶B in 30 min, R_t_ = 22.5 min) the glyco-lipopeptide HER-GLP-1 was obtained as pure lyophilized powder (5 mg, 15% yield). Analytical data: R_t_ = 11.8 min (C_18_ analytical column, linear gradient: 95∶5 to 0∶100 A∶B in 15 min), ES-MS (positive mode) for C_237_H_391_N_58_O_73_S_2_ 5281.8, found 5282.9.

### Immunization and serum preparation

Female B10.D1 mice 4 to 5 weeks old were purchased from Jackson Laboratory (Bar Harbor, ME) and immunized subcutaneously at the base of the tail either with (*i*) linear HER- GLP-1 (50 µM/mouse in 100µl of PBS) or with (*ii*) branched HER-GLP-2 (50 µM/mouse in 100µl of PBS) or with (*iii*) HER- (50 µM/mouse in 100µl of PBS) or (*iv*) PBS alone, three times at 14 day intervals. Ten days after the last immunization, mice were bled from the ocular venus plexus. Serum were collected by centrifugation for 5 min at 1250 RPM and stored at −80°C.

### Antibodies

Streptavidin coated 96-well ELISA plates were purchased from NUNC. MultiScreen HTS™ IP plates for ELISpot assays were purchased from Millipore. Goat anti-mouse IgG-HRP was purchased from Chemicon. Goat anti-mouse IgG-FITC was purchased from Sigma. TMB substrate reagent set was purchased from BD Biosciences.

### ELISA and ELISpot assay

Immune and non-immune mouse sera were tested for anti-RAFT antibodies by using direct ELISA, as we recently described [21], [82]. ELISpot assay was performed by using cytokine ELISpot pair kit from BD PharMingen, San Diego, CA, as we recently described [21].

### Cell lines

MCF-7, the human breast cancer cell lines were obtained from the American Type Culture Collection (Manassas, VA) and grown to 90% confluence in Modified Dulbecco's medium, as we recently reported [5], [6], [21], [105]. The NT2 neu-expressing tumor line derived from spontaneously arising mammary tumors excised from *neu*-N mice was used in experiment of tumor growth inhibition [106]. NT2 cells express stable neu and MHC class I, as previously described [106].

### Generation of DCs

Bone marrow-derived DCs were generated using a modified version of our previously described protocol [85]. Briefly, a total of 2×10^6^ bone marrow cells were cultured in tissue dishes containing 10 ml of RPMI 1640 supplemented with 2 mM glutamine, 1% nonessential amino acids (Gibco-BRL), 10% FCS, 50 ng/ml of GM-CSF and 50 ng/ml IL-4 (PeproTech Inc). Cells were feed with fresh medium supplemented with 25 ng/ml of GM-CSF and 25 ng/ml of IL-4 every 72 h. After 7 days of culture, this protocol yielded 50×10^6^–60×10^6^ cells with 70–90% of the non-adherent cells displaying the typical morphology of DC. This was routinely confirmed by FACS analysis of CD11c and DEC-205 surface markers of DC.

### Dendritic cell surface and cytosolic uptake assay

One million DC were suspended in at 37°C pre-warmed RPMI 1640 medium containing 5% FCS and incubated for 2 h with 0.1, 1, 10 and 100 ng/ml of linear HER- GLP-1 or branched HER- GLP-2 constructs labeled with Alexa Fluor®488. Cells were harvested every 10 min, washed in cold FACS® buffer and stained with 1 ug/ml of FITC-labeled anti-CD11c, anti-Mac-1 (CD11b) (PharMingen, San Diego, CA). Cells were then acquired using a FACSCalibur® with two excitation laser sources and analyzed with CellQuest® software (Becton Dickinson, San Jose, CA), as we previously described [85]. Confocal microscopy was used to visualize the cell surface labeling and to follow the intracellular delivery of linear HER- GLP-1 or branched HER- GLP-2 constructs, as we previously described [85]. The PE or FITC conjugated secondary antibodies were used to assess the surface expression of CD80 (clone 10–10A1, IgG), CD86 (clone GL1, IgG2a) and FITC-MHC class II (clone M5/114.15.2, IgG2b, *k*) (PharMingen), respectively. IgG isotype-matched irrelevant mAbs were used as controls. After staining, 20,000 events were acquired on a Becton Dickinson (Mountain View, CA) FACSCalibur® and the expressions of the markers of maturation were analyzed on CD11c-gated cells using CellQuest software.

Endocytosis assay showing the uptake of Alexa Fluor 488-labeld HER-GP and HER-GLPs by DCs was measured for 120 min at both 4C° and 37C° respectively. Cells were washed three times with phosphate-buffered saline containing 1% bovine serum albumin and total uptake of Alexa Fluor 488-labeld was measured by FACS. The data are expressed by the difference in geometric mean after subtracting the values obtained at 4C° from the values obtained at 37C°. This formula determines the amount of HER-GP and HER-GLPs that is actively internalized.

### Flow Cytometry

200 µl of 4×10^5^ cancer cells were incubated with 10ul of (1∶250 dilution) immunized or non-immune serum for 30 min. at 4°C in FACS staining buffer (PBS containing 5% fetal calf serum and 0.1% sodium azide). Cells were washed two times with buffer followed by centrifugation for 5 min at 1250 RPM and 5°C after each wash. Cell pellets were suspended in 200 µl of FACS staining buffer and incubated with 10µl (undiluted) of secondary antibody (anti-mouse IgG-FITC) for 30 min. at 4°C. Cells were washed two times as above. The final cell pellets were suspended in 250 µl of FACS staining buffer and data acquired immediately on a FACScan flow cytometer (Becton Dickinson, Mountain View, CA, USA) and analyzed with CellQuest software (Becton Dickinson).

### Blocking TLR-2 receptors on DCs

Blocking of TLR-2 receptors on DCs were performed on day 4 after the initiation of the culture from bone marrow derived cells, as previously described [85]. Anti-TLR-2 mAb (*e*Bioscience) or an IgG1 isotype control mAb (10 µg/ml) was added to DCs 30 min before the addition of the peptide or lipopeptide. Cells were harvested 48 h later and counted directly in ELISpot Assay.

### Monensin antigen-processing inhibitors

Dendritic cells were first treated with by brefeldin A, Epoxomycin or Monensin, followed by the addition of either linear HER-GLP-1, branched HER-GLP-2 or parent non-lipidated HER-GP construct and incubated overnight with HER_420–429_-specific CD8^+^ T cells. When used, brefeldin A (Sigma) or epoxomycin (Alexis Biochemicals) was added 1 h before the addition of linear HER-GLP-1, branched HER-GLP-2 or parent non-lipidated HER-GP constructs at concentrations of 10 µg/ml and 10 µM, respectively, and then diluted at 2 µg/ml and 2 µM for the overnight incubation [26], [27]. Monensin (Sigma) was added only 10 min after linear HER-GLP-1, branched HER-GLP-2 or parent non-lipidated HER-GP addition (in an attempt to avoid preventing their endocytosis) at a concentration of 50 µM. In all cases, after overnight incubation, DCs were then washed and added to the HER_420–429_-specific CD8^+^ T cells (ratio, 1∶1) in the presence of brefeldin A (10 µg/ml) for 5 h at 37°C. CD8^+^ T cells incubated in the presence of non-treated but pulsed DCs were used as positive controls. After inhibition CD8^+^ T cells were assessed in ELISpot for IFN-γ production as indicated above.

### Tumor immunotherapy

Ten mice in each experimental group were inoculated s.c. in the upper back with 1×10^5^ NT2 cells/mouse. Local tumor diameter was measured with calipers. Starting 8–10 days later, when the tumor reached 3–4 mm in diameter, mice were immunized sc. four times at 7-day intervals with GLP-1 and/orGLP-2 or control PBS (Mock) on days 0, 7 and 14 and 21, as described above. Tumor diameter and survival were recorded. The length, width and height of each tumor were measured using a digital slide caliper. Tumor volume was calculated by the formula: π/6 × length × width × height.

### Statistical analysis

Statistical differences in tumor sizes between groups of mice were determined by one-way ANHER. Significance of survival plots was done with Kaplan-Meier survival platform. For both analyses, we used the JMP statistics software (SAS Institute).
